# Magnetic core‐shell Fe_3_O_4_@TiO_2_ nanocomposites for broad spectrum antibacterial applications

**DOI:** 10.1049/nbt2.12017

**Published:** 2021-04-20

**Authors:** Nisha Rani, Brijnandan S. Dehiya

**Affiliations:** ^1^ Department of (MSN) Materials Science and Nanotechnology Deenbandhu Chhotu Ram University of Science and Technology (DCRUST) Murthal Haryana India

## Abstract

The authors have synthesised a core‐shell Fe_3_O_4_@TiO_2_ nanocomposite consisting of Fe_3_O_4_ as a magnetic core, and TiO_2_ as its external shell. The TiO_2_ shell is primarily intended for use as a biocompatible and antimicrobial carrier for drug delivery and possible other applications such as wastewater remediation purposes because of its known antibacterial and photocatalytic properties. The magnetic core enables quick and easy concentration and separation of nanoparticles. The magnetite nanoparticles were synthesized by a hydrothermal route using ferric chloride as a single‐source precursor. The magnetite nanoparticles were then coated with titanium dioxide using titanium butoxide as a precursor. The core‐shell Fe_3_O_4_@TiO_2_ nanostructure particles were characterized by XRD, UV spectroscopy, and FT‐IR, TEM, and VSM techniques. The saturation magnetization of Fe_3_O_4_ nanoparticles was significantly reduced from 74.2 to 13.7 emu/g after the TiO_2_ coating. The antibacterial studies of magnetic nanoparticles and the titania‐coated magnetic nanocomposite were carried out against gram+ve, and gram–ve bacteria (*Staphylococcus aureus, Pseudomonas aeruginosa, Shigella flexneri* *, Escherichia coli*, and *Salmonella typhi*) using well diffusion technique. The inhibition zone for *E. coli* (17 mm after 24 h) was higher than the other bacterial strains; nevertheless, both the uncoated and TiO_2_‐coated magnetite nanocomposites showed admirable antibacterial activity against each of the above bacterial strains.

## 1 INTRODUCTION

Metal oxide nanostructured particles are of great significance in the field of antibacterial studies because of large surface areas with extremely active sites. A very discrete type of binary‐metal oxide having good magnetization and unique biocompatibility is magnetite (Fe_3_O_4_) especially with crystal size on the nanoscale. Fe_3_O_4_ nanoparticles have been widely used because of some of its excellent optical and magnetic properties [1, 2, 3, 4]. In addition to these, plural cationic oxidation states in Fe_3_O_4_ nanoparticles provide an extra edge over other materials for physicochemical applications [5, 6, 7]. Various methods have been used by researchers to synthesize high‐quality monodisperse magnetic nanoparticles such as chemical co‐precipitation, solvothermal, hydrothermal, microemulsion, thermolysis, decomposition of precursors, sol‐gel, and polyol methods [8, 9, 10, 11, 12, 13, 14, 15, 16]. The agglomeration of crystals is an undesirable occurrence during the synthesis of magnetic nanoparticles. Functionalization and coating of nucleated crystals with ligands can prevent the agglomeration of nanoparticles. Nanoparticles can also have a stronger interaction with the biological cells' surface because of their high surface area to volume ratio compared with bulkier crystals [17]. Some nanoparticles like ZnO, CdSe, TiO_2_, ZnS, and SiO_2_ have shown considerable antibacterial/antimicrobial activity, and selective toxicity in biological systems has previously reported [18]. The antibacterial activity of TiO_2_ has been attributed mainly to its capability to activate free hydroxyl radicals (OH^−^) under the action of sunlight/ultraviolet radiation [19].

Combining Fe_3_O_4_ and TiO_2_ nanoparticles to achieve the easy recovery and recycling of TiO_2_ nanoparticles permits one solution of remediating the contamination of the environment by wastewater. The synthesized nanocomposites could also be easily removed using a magnet after antibacterial application allowing for repeated use of the nanomaterial. The biochemical interaction that occurs between the nanoparticles and the microbes has been attributed to the positive charge on nanoparticles and the negative charge on the outer cell walls of the microbes, which results in the oxidation of microbes and quick death [20]. Cell lysis mechanism also involves the reaction between the ions generated by nanostructures particles and protein on the bacterial cell [21]. The mechanism of antibacterial action by the nanoparticles thus is also through the oxidative stress due to Reactive Oxygen Species (ROS) that are generated by the nanoparticles [22, 23]. The singlet oxygen produced could also damage the proteins or the DNA in the bacteria and thus lead to the bacteria’s degradation. Kim et al. studied the generation of H_2_O_2_ when Fe^2+^reacted with dissolved oxygen. The reaction between the Fe^2+^and H_2_O_2_ also creates the ^•^OH radical, which harms the biological macromolecules’ structures [24].

Magnetite (Fe_3_O_4_) nanoparticles were prepared by using a single precursor. Afterwards, the magnetite nanoparticles were coated with titania using titanium butoxide as a precursor. We have explored here the effect of these Fe_3_O_4_@TiO_2_ nanostructured composite particles as antibacterial agents on five different pathogen bacteria such as *Staphylococcus aureus, Pseudomonas aeruginosa, Shigella flexneri, Escherichia coli,* and *Salmonella typhi*. A unique attraction for this study was that these nanocomposites can be quickly concentrated, recovered, and recycled using a simple magnetic separation technique, in‐vitro and in‐vivo.

## 2 EXPERIMENTAL DESIGN

### 2.1 Materials

Iron (III) chloride, NH_4_Ac, ethylene glycol (EG), tetracycline, polyvinyl pyrrolidone, and LB agar were all purchased commercially (MERCK). Titanium butoxide was purchased from Sigma Aldrich. Five bacterial species, that is, *S. aureus, P. aeruginosa, Shigella flexneri, E. coli*, and *S. typhi,* were acquired locally from the Department of Biotechnology, DCRUST University, Sonepat, India.

### 2.2 Synthesis route

#### 2.2.1 Synthesis of Fe_3_O_4_ nanoparticles

0.6 M of Iron(III) chloride, NH_4_Ac (1.5 M), and PVP (0.25 M) were put in 40 ml of ethylene glycol under magnetic stirring. After 3 h of mixing, the solution was shifted into an autoclave for hydrothermal action at 200°C for 20 h. Finally, the autoclave was cooled down, and the black precipitate was taken apart with a magnet. The precipitate was rinsed with ethanol and dehydrated at 70°C for 7–8 h [25].

#### 2.2.2 Synthesis of Fe_3_O_4_@TiO_2_ nanoparticles

0.5 g of Fe_3_O_4_ nanostructure particles were put in 10 ml of ethanol with a concentrated 0.50 ml ammonia solution under ultrasound for 20 min. Afterwards, 5 ml of TBOT in ethanol (40 ml) was put into the solution drop wise under the stirrer. Following 2 h stirring at room temperature, the ultrasonication was done for 2 h. The suspension was put into an autoclave for hydrothermal action at 200°C for 20 h. The sample was taken apart by a magnet, rinsed with ethanol, and dried at 70°C.

### 2.3 Antibacterial activities screening using the well diffusion method

250 ml of LB agar was prepared. The media was poured into five Petri plates. Once the medium was solidified, 500 μl of cultures of age 18–24 h were spread on solidified media in all Petri plates. Then uniform holes were made, and 50 mg/ml concentration of nanoparticles was poured into the holes. The enclosed parafilm plates were set aside into the incubator at 37^o^C for 24 h. A negative control (ethanol) and positive control (15 μl of 10 mg/ml tetracycline) was used. The zone of inhibition was noted in mm [26].

## 3 RESULTS AND DISCUSSION

### 3.1 XRD characterisation

#### 3.1.1 Fe_3_O_4_ nanoparticles

**FIGURE 1:**
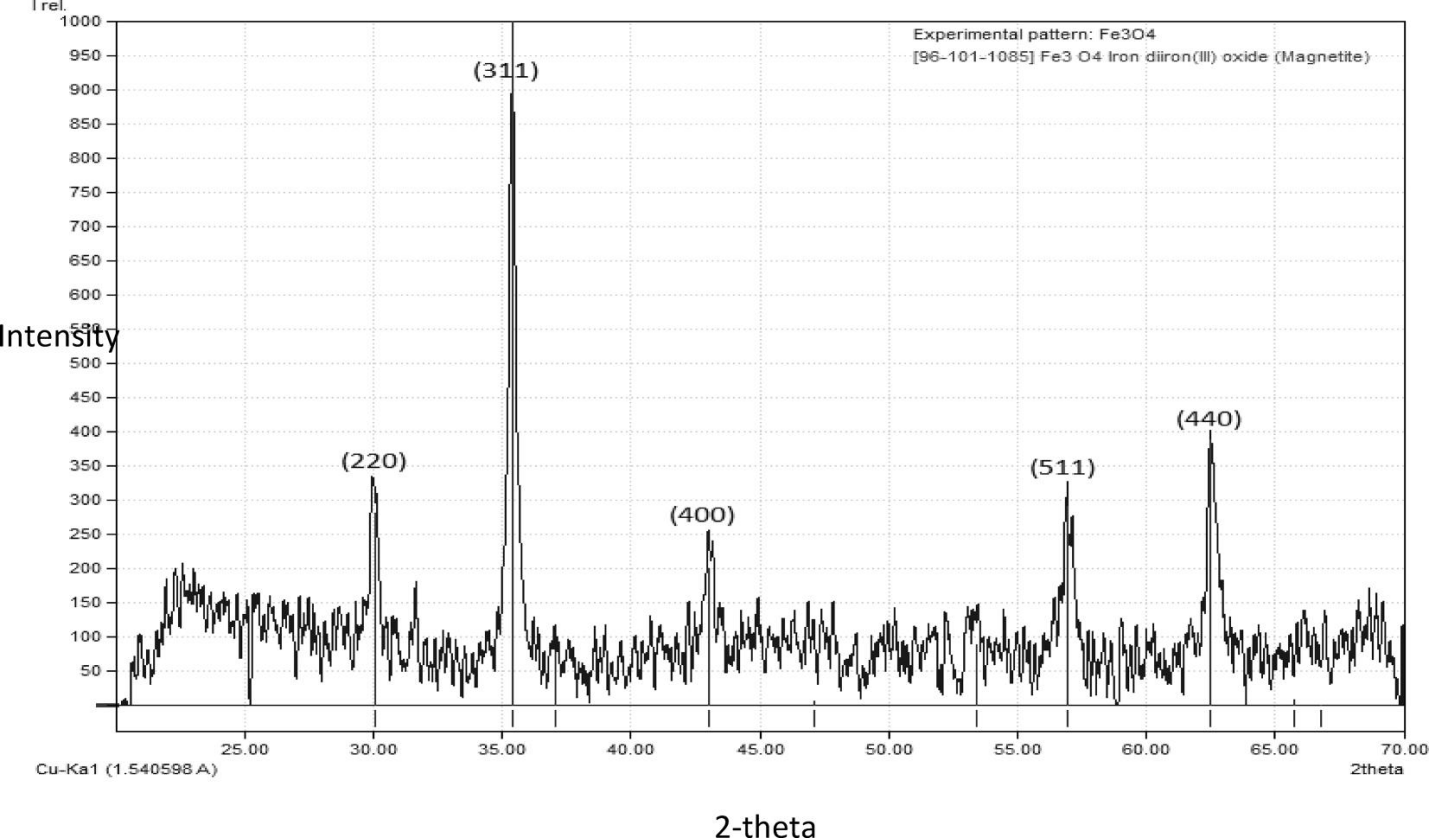
The X‐ray diffraction graph of the chemically synthesized magnetite nanoparticles is shown

Figure 1 shows the X‐ray diffraction pattern from the magnetite synthesized by the chemical co‐precipitation process. These nanoparticles have been investigated using XRD. The XRD chart of magnetite nanostructure particles corresponds to a sequence of peaks at 2θ of 30.1^o^, 35.5^o^, 43.2^o^, 53.4°, 57.2°, and 62.8°, which are associated to the (220), (311), (400), (422), (511), and (440) planes of magnetite, respectively (Crystallography Open Database PDF No. 96‐101‐1085) [27].

The highly crystalline structure of the magnetite is shown by the sharp peaks of the XRD graph in Figure 1.

#### 3.1.2 Fe_3_O_4_@TiO_2_ nanoparticles

**FIGURE 2:**
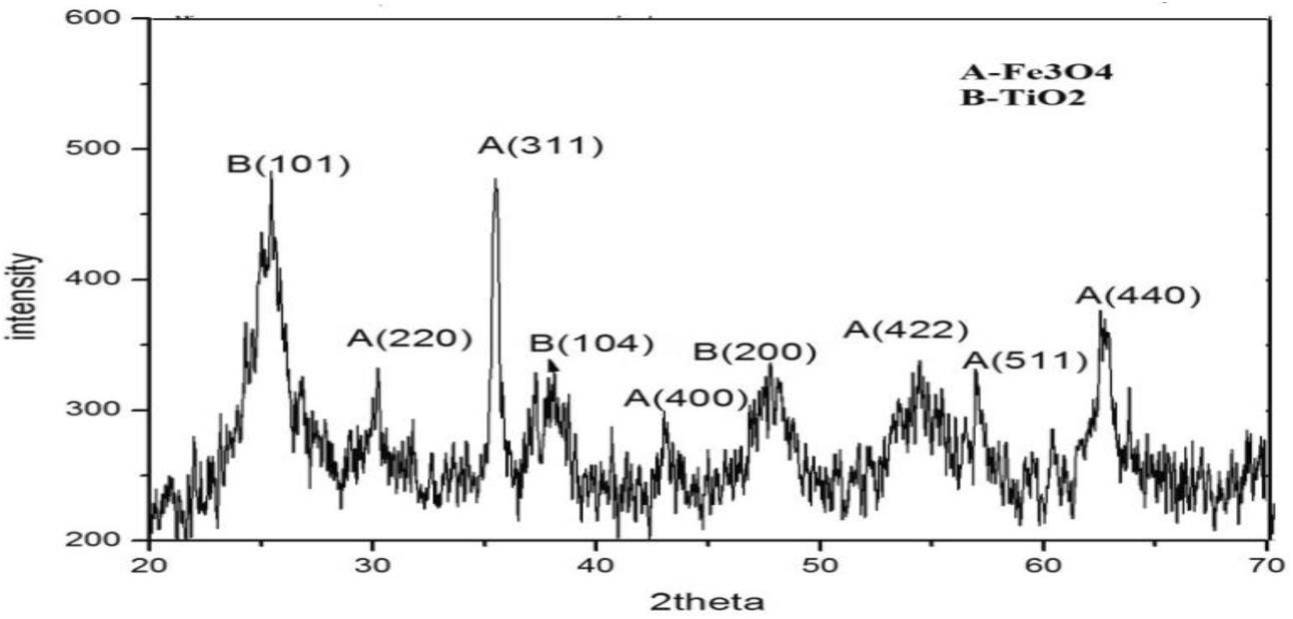
The X‐ray diffraction graph of Fe_3_O_4_@TiO_2_ core‐shell nanocomposite is shown here

The XRD graph of these nanocomposites shows peaks at 2θ of 25.3^o^, 37.8^o^, 48.2^o^, that is corresponding to the reflection of (101), (104), and (200) planes of TiO_2_ nanostructures particles, respectively [28]. Some peaks of Fe_3_O_4_ cores and the intense peaks of anatase form of titania are shown in the XRD graph, confirming the presence of TiO_2_ coating on magnetite nanostructured particles (Figure 2), which reduces the relative peak intensity of the magnetite phase in the diffraction pattern.

### 3.2 FTIR

#### 3.2.1 Fe_3_O_4_ nanoparticles

The FTIR spectrum represents hydroxyl group, which is recognized as absorption at 3429 cm^−1^. The band at 2929 cm^−1^ can be attributed to the CH_2_ stretching bond.

**FIGURE 3:**
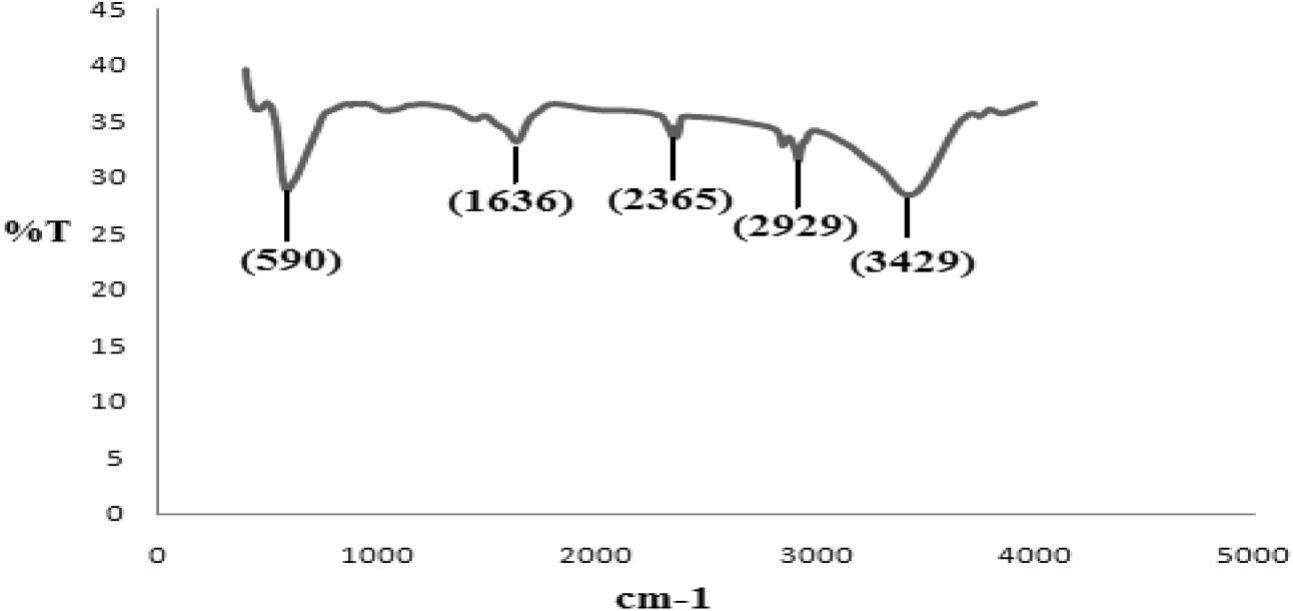
FTIR spectrum of the uncoated Fe_3_O_4_ nanoparticles is shown here

The band at 2365 cm^−1^ is due to CO_2_ in the atmosphere. In the spectrum, absorption at 590 cm^−1^ shows a Fe‐O bond. The peak at 1636 cm^−1^ is reported as an amino group [29] (Figure 3).

#### 3.2.2 Fe_3_O_4_@TiO_2_ nanoparticles

The FTIR spectrum showed a band at 3390 cm^−1^, which is recognized as an O‐H bond. The band at 2360 cm^−1^ is ascribed to the presence of CO_2_ in the chamber atmosphere.

**FIGURE 4:**
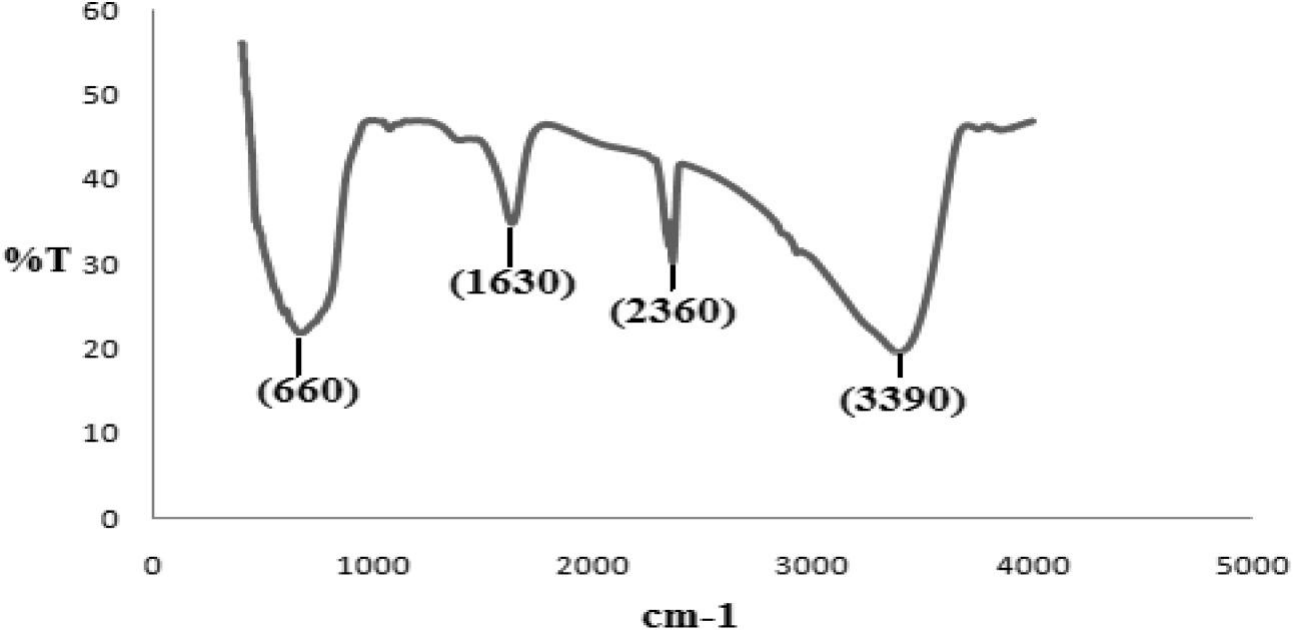
The FTIR spectrum of Fe_3_O_4_@TiO_2_ nanoparticles is shown here

The band at 1630 cm^−1^ is recognized as NH bending. The band at 660 cm^−1^ is assigned to the Ti‐O‐Ti stretching vibrations [30] (Figure 4).

### 3.3 UV characterization

**FIGURE 5:**
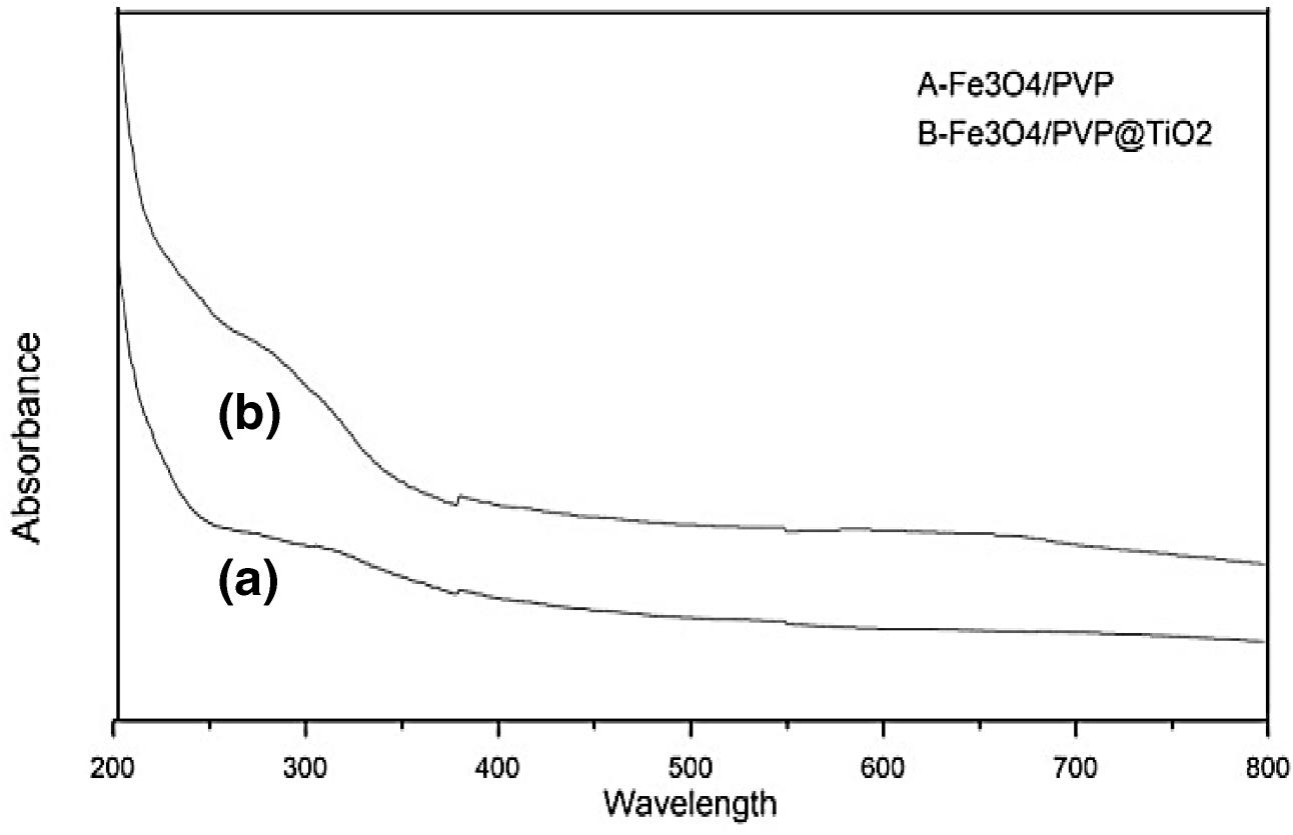
UV‐Vis absorbance spectra of the uncoated Fe_3_O_4_ nanoparticles (a) and of the Fe_3_O_4_@TiO_2_ nanocomposite (b)

Figure 5 shows the absorption spectrum of magnetite and titania‐coated Fe_3_O_4_ nanocomposite. When titania nanoparticles were coated on the Fe_3_O_4_ nanoparticles, the absorption peak(s) was shifted into the visible region.

### 3.4 TEM characterization

**FIGURE 6:**
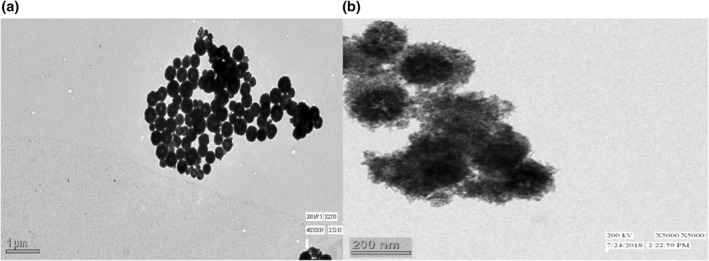
(a) TEM images of magnetite nanoparticles (b) TEM images of titania‐coated magnetite nanocomposite structures

Both single‐phase Fe_3_O_4_ nanoparticles and Fe_3_O_4_@TiO_2_ core‐shell nanostructures, were synthesized using a single precursor by the hydrothermal process. A lot of nanostructured crystalline granules of titania appear to enclose a magnetite core. The titanium dioxide is present as the externally deposited film on the Fe_3_O_4_ nanostructure particles, creating the Fe_3_O_4_@TiO_2_ nanostructure [31] (Figure 6).

### 3.5 VSM characterization

**FIGURE 7:**
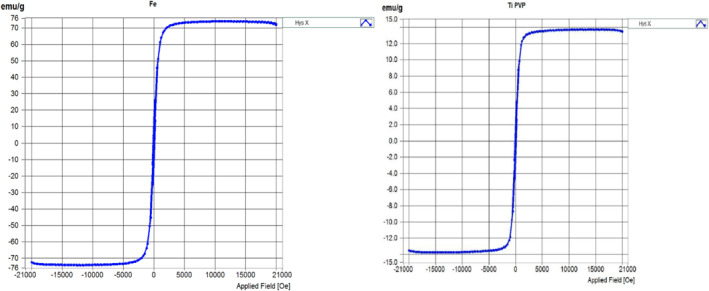
Saturation magnetization of magnetite and titania‐coated magnetic nanoparticles

The magnetic features of Fe_3_O_4_ nanoparticles and Fe_3_O_4_@TiO_2_ nanostructure composites have been studied using the VSM technique (Figure 7).

The saturation magnetization (M_s_) of magnetite nanostructure particles and Fe_3_O_4_@TiO_2_ nanostructure composites were 74.268 emu/g and 13.755 emu/g, respectively. The saturation magnetization (M_s_) reduced because of the non‐magnetic TiO_2_ outside film on nanoparticles (Table 1).

**TABLE 1:** The magnetic measurements of the uncoated and TiO_2_‐coated magnetite samples

S. No.	Sample Name	Saturation Magnetization (M_s_) (emu/g)	Coercivity (H_c_) (Oe)	Remanence (M_r_) (emu/g)
1	Fe_3_O_4_/PVP	74.268	69.217	7.120
2	Fe_3_O_4_/PVP@TiO_2_	13.755	69.366	1.316

**TABLE 2:** The diameter of the inhibition zone for Fe_3_O_4_@TiO_2_ nanocomposites against different extracts

S. No.	Strain	Inhibition Zone (mm) (Fe_3_O_4_)	Inhibition Zone (mm) (Fe_3_O_4_@TiO_2_)
1	*Staphylococcus aureus* (gram +)	13.33 ± 0.58	16.17 ± 0.29
2	*Escherichia coli* (gram −)	15.50 ± 0.50	16.50 ± 0.50
3	*Shigella flexneri* (gram −)	11.17 ± 0.76	14.83 ± 0.76
4	*Pseudomonas aeruginosa* (gram −)	10.50 ± 0.50	15.83 ± 0.29
5	*Salmonella typhi* (gram −)	12.67 ± 0.29	14.33 ± 0.76

### 3.6 Antibacterial activities

**FIGURE 8:**
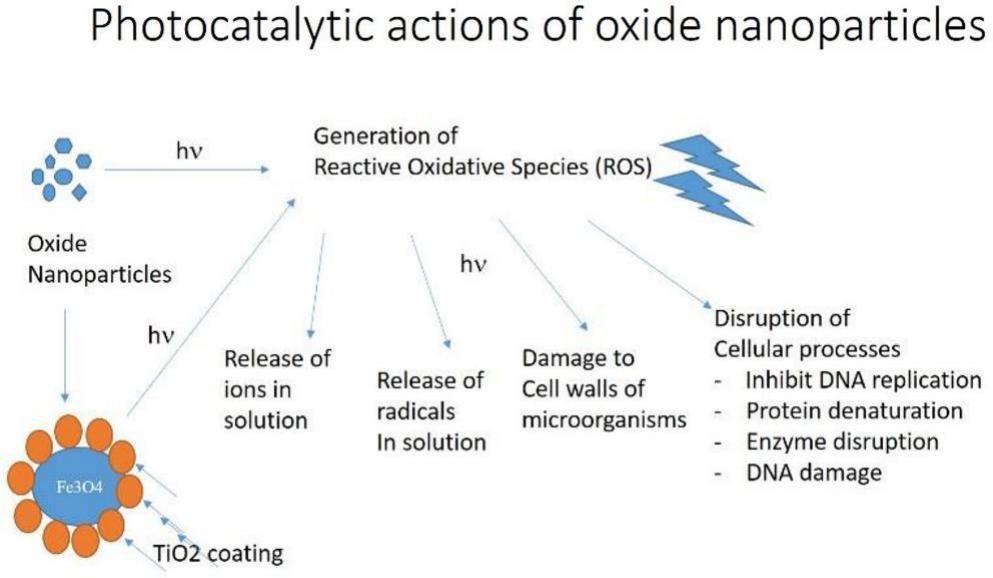
The mechanism of interaction of oxide NPs with bacterial/microbial cells

TiO_2_‐coated magnetite exhibited bactericidal activity in resistance to both gram *+*ve (positive) and gram *–*ve (negative) bacteria. The antibacterial activity by the core‐shell nanocomposite is likely due to the oxidative stress produced by reactive oxygen species (ROS) which include superoxide radical (O^−2^), hydroxyl radical (^–^OH), and also hydrogen peroxide (H_2_O_2_) (Figure 8). Collin [32] showed how hydrogen peroxide (H_2_O_2_) and other ROS were generated when Fe^2+^/Fe^3+^reacted with oxygen. The biochemical interaction occurs between H_2_O_2_ and membrane proteins or between the substance formed due to magnetite nanoparticles and bacteria's external bilayer. Then H_2_O_2_ enters the outer bilayer of bacteria and destroys them [33]. The various oxidation and reduction reactions occur when Fe_3_O_4_@TiO_2_ nanocomposites disperse within the media, known as the Fenton reaction in case of the iron ionic species. These reactions generate different reactive oxygen species because of the Fe^3+^ and Fe^2+^ present in magnetite [34, 35]. It has also been reported that Fe^3+^ doping of TiO_2_ reduces agglomeration resulting in high photocatalytic efficiency and a reduced bandgap of about 2.6 eV [36]. Several oxidation‐reduction and photocatalytic reactions can occur simultaneously in this combination:

(1) $$O_{2}+e^{-}\;\to \;{O^{-}}_{2}+e^{-}+{2H}^{+}\to H_{2}{\text{O}}_{2}+{\text{e}}^{-}\;\to \;2\text{HO}●$$

(2) $${\text{Fe}}^{3+}+{\text{H}}_{2}{\text{O}}_{2}\;\to \;{\text{Fe}}^{2+\;}+{\text{OH}}^{+}+\text{OH}●$$

(3) $${\text{Fe}}^{3+}+{\text{H}}_{2}{\text{O}}_{2}\;\to \;{\text{Fe}}^{2+\;}+{\text{HO}}_{2}{●}^{\;}+{\text{H}}^{+}$$

(4) $${\text{Fe}}^{2+}{+\text{O}}_{2}\;↔\;{\text{Fe}}^{3+\;}+{\text{O}}_{2}^{●}$$

(5) $${\text{O}}_{2}^{●}+{\text{O}}_{2}^{●}+{2\text{H}}^{+}↔{\text{O}}_{2}+{\text{H}}_{2}{\text{O}}_{2}$$

(6) $${\text{H}}_{2}{\text{O}}_{2}{+\text{Fe}}^{2+}↔{}^{●}\text{OH}+\text{HO}-+{\text{Fe}}^{3+}$$

(7) $${\text{TiO}}_{2}+\text{h}\nu \to {{\text{h}}^{+}}_{\text{VB}}+{{\text{e}}^{-}}_{\text{CB}}$$

(8) $${\text{H}}_{2}{\text{O}}_{2}+{{\text{h}}^{+}}_{\text{VB}}\to \text{H}+{\text{OH}}^{●}$$

OH^●^ and HO_2_^●^ produced in the reaction are reactive free radicals. Magnetite (Fe_3_O_4_) NPs can slowly be oxidized to maghemite (*γ*‐Fe_2_O_3_). This oxidation is a critical part of the origin of oxidative stress to the cell of bacteria, resulting in the bacterial cell's death.

**FIGURE 9:**
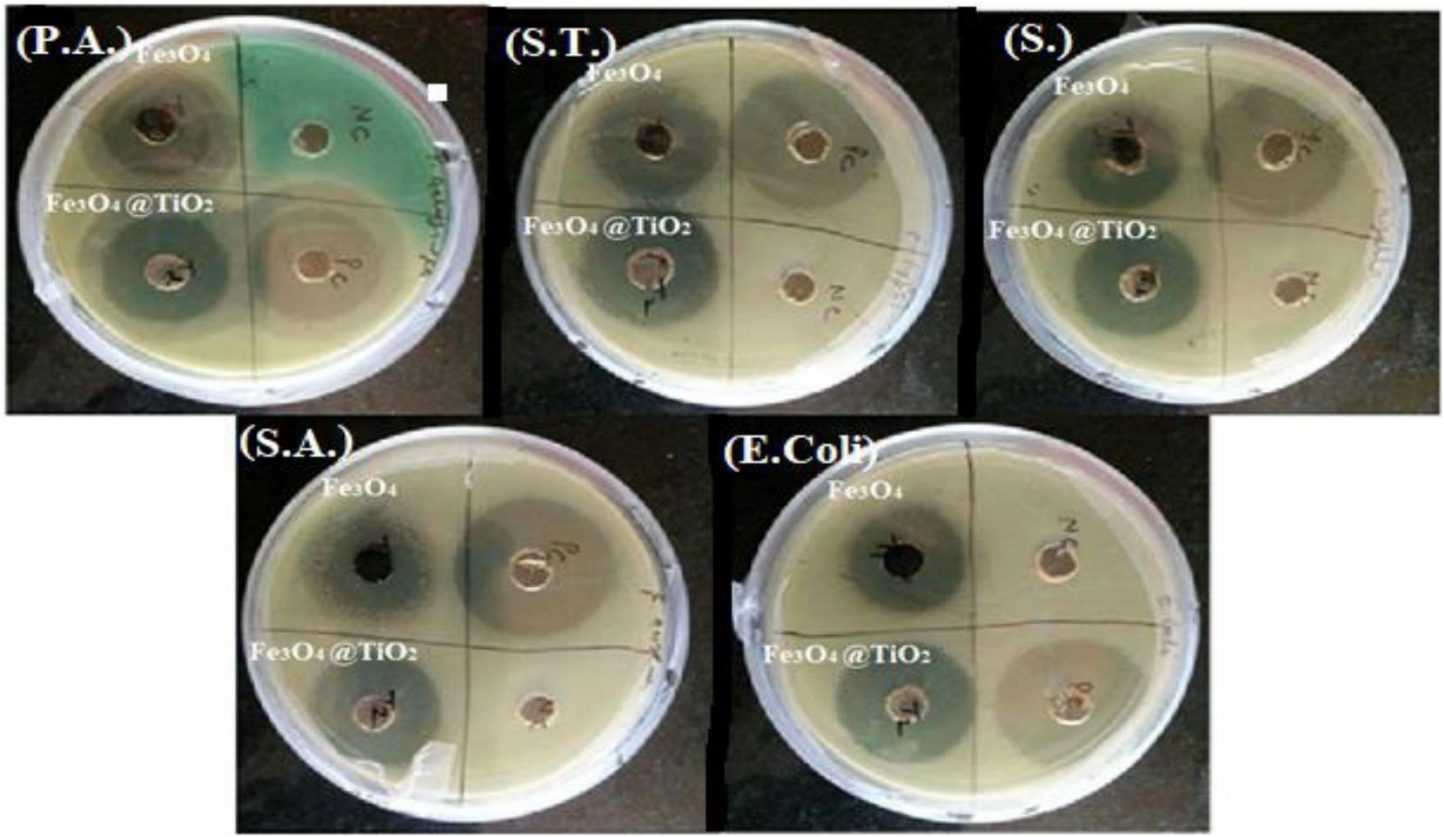
Antibacterial activity of both Fe_3_O_4_ nanoparticles and Fe_3_O_4_@TiO_2_ nanoparticles against different extracts, that is, P.A. – *P. aeruginosa*, S.T*. – S. typhi*, S. – *Shigella flexneri,* S.A. – *S. aureus*, *E. coli*

Table 2 and figure 9 depict the effect of the uncoated and coated magnetite nanoparticles on the five different species of microorganisms. The terms ‘nc’ and ‘pc’ in Figure 9 refer to negative control and positive control, respectively. The antibacterial actions of both types of the nanoparticles are clearly very significant, with TiO_2_‐coated particles providing superior antibacterial action. The strongest effect was observed on *E. coli* bacteria for both the types of nanomaterials.

#### 3.6.1 Antibacterial index of magnetite nanoparticles

**FIGURE 10:**
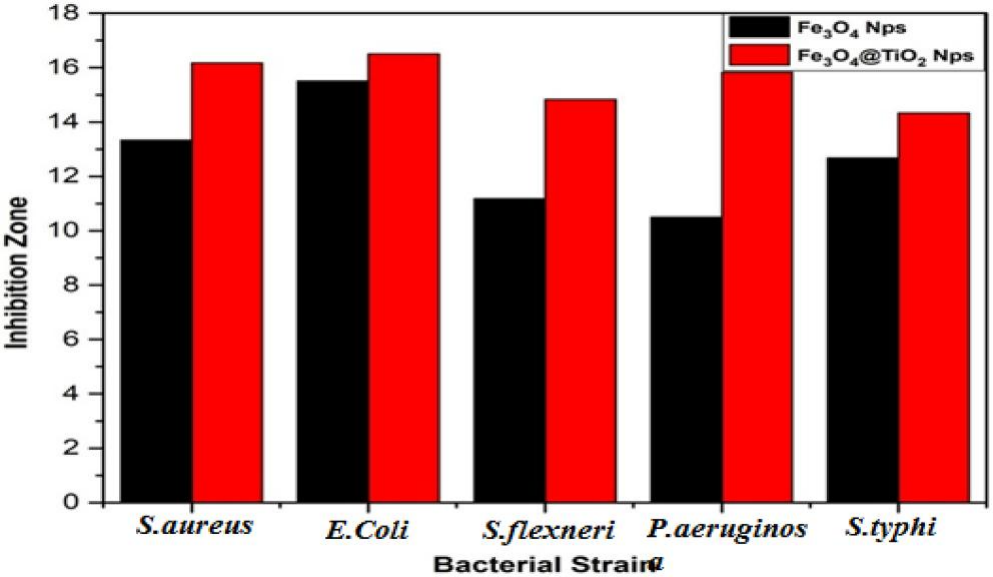
Antibacterial index of magnetite nanoparticles and titania‐coated magnetic nanocomposites are shown here

The antibacterial index of magnetite nanostructures particles and Fe_3_O_4_@TiO_2_ nanocomposite was shown in Figure 10. The Fe_3_O_4_@TiO_2_ nanocomposite exhibited a better bactericidal activity for *E. coli* as compared with other bacterial strains.

The magnetite nanoparticles, as core materials, showed good bactericidal activity. The maximum antibacterial activity was observed for TiO_2_‐coated nanoparticles because of its inherent antibacterial properties [37]. The core‐shell Fe_3_O_4_@TiO_2_ nanocomposite showed a better antibacterial effect than Fe_3_O_4_ nanoparticles. The activity of Fe_3_O_4_ nanoparticles to inactivate bacterial strains was improved significantly after coating with TiO_2_.

The core‐shell Fe_3_O_4_@TiO_2_ nanocomposite could be used for drug delivery because of the inherent antibacterial property of TiO_2_ and the magnetic property of magnetite nanoparticles. The ability to kill cancer cells has also been previously studied [38, 39]. The TiO_2_ nanoparticles have the capability to both oxidize the pollutants and kill the microorganisms [40]. The antibacterial effect of core‐shell Fe_3_O_4_@TiO_2_ nanocomposite was studied, which combined the treatment of microbial contamination and magnetic separation property of magnetite, synthesized. The antibacterial activity of TiO_2_ nanoparticles using as a shell was better than that reported in previous studies [41, 42].

## 4 CONCLUSION

Magnetite superparamagnetic nanostructures particles were fabricated using a single precursor by the hydrothermal method and by using a reducing agent. The Fe_3_O_4_ nanostructured particles were coated with titania using a separate hydrothermal process. The effect of the TiO_2_ shell in the core‐shell Fe_3_O_4_@TiO_2_ nanocomposite as a broad‐spectrum antibacterial agent was investigated, in comparison to the similar effect of the uncoated core magnetite itself. The antibacterial effects of magnetite core and the titania shell were studied on five bacteria strains: *S. aureus*, *P. aeruginosa*, *Shigella flexneri*, *E. coli*, and *S. typhi*. The Fe_3_O_4_@TiO_2_ nanocomposites showed superior antibacterial action on each bacterium, while the uncoated magnetite’s effect was significantly lesser in comparison. The TiO_2_‐coated magnetite nanoparticles revealed better and more effective antibacterial activity against the *E. coli* strain of bacteria than the other strains. It can be concluded that Fe_3_O_4_@TiO_2_ is a very active antibacterial agent, as verified by the large diameter of the inhibition zone. The results indicated that nanocomposites synthesized in this work could also be suitable materials for drug delivery applications. These can also be practical and cost‐effective agents in cleaning a microbe‐polluted water environment where the magnetic core lends itself to easy recycling. The results reveal that bacterial illness could be treated by core‐shell Fe_3_O_4_@TiO_2_ nanostructure particles employing TiO_2_ as a shell due to its inherent antibacterial properties. Fe_3_O_4_@TiO_2_ nanocomposites could be efficient and recyclable antibacterial agents because of their magnetic, anti‐bacterial and photocatalytic features.
